# Supervised Toothbrushing Programmes: Understanding Barriers and Facilitators to Implementation

**DOI:** 10.1111/cdoe.13026

**Published:** 2025-01-29

**Authors:** Kara A. Gray‐Burrows, Sarab El‐Yousfi, Kristian Hudson, Samantha Watt, Ellen Lloyd, Hanin El Shuwihdi, Tom Broomhead, Peter F. Day, Zoe Marshman

**Affiliations:** ^1^ School of Dentistry University of Leeds Leeds UK; ^2^ School of Clinical Dentistry University of Sheffield Sheffield UK; ^3^ Improvement Academy Bradford Institute for Health Research Bradford UK; ^4^ Community Dental Service Bradford District Care NHS Foundation Trust Bradford UK

**Keywords:** child, focus groups, implementation science, nursery, oral health, schools, stakeholder participation, toothbrushing

## Abstract

**Objectives:**

Supervised toothbrushing programmes (STPs), whereby children brush their teeth at nursery or school with a fluoride toothpaste under staff supervision, are a clinically and cost‐effective intervention to reduce dental caries. However, uptake is varied, and the reasons unknown. The aim was to use an implementation science approach to explore the perspectives of key stakeholders on the barriers and facilitators at each level of implementation of STPs.

**Methods:**

This qualitative study involved individual interviews and focus groups with a purposive sample of stakeholders involved at all levels of implementation of STPs: (1) policymakers; (2) providers of STPs; (3) nursery/school staff; (4) parents/carers; and (5) children (aged 2‐6 years old) across England. Data collection and analysis were guided by the Consolidated Framework for Implementation Research (CFIR).

**Results:**

A total of 159 stakeholders were interviewed (40 individual interviews and 17 focus groups) across all levels of implementation. Barriers and facilitators to STP implementation were identified across 35 of the 39 CFIR constructs. Four themes were identified that determined STP implementation: (1) acceptability of STPs; (2) external ‘make or break’ conditions; (3) the importance of engagement across the system; and (4) desire for centralised support.

**Conclusions:**

This is the first study to qualitatively explore the barriers and facilitators to STP at all levels of implementation underpinned by an implementation science framework. The findings have strong implications for policymakers who wish to implement STPs, highlighting the need for careful consideration of the adaptability of the programmes, the role of formal and informal engagement systems, and the need for centralised support. This work has facilitated the co‐design and piloting of a supervised toothbrushing implementation toolkit, which provides a central hub of resources and good practice to optimise implementation of STPs at scale.

## 1 Introduction

Dental caries is the most common childhood health condition, affecting 600 million worldwide and placing a substantial burden on health and the economy [1]. Dental caries causes pain and suffering, affecting what children eat, their speech, quality of life, self‐esteem, social confidence and has a wider societal impact on school readiness and attendance. In England, nearly a quarter of 5‐year‐olds have dental caries with the extraction of decayed teeth being the most common reason for hospital admissions [2], costing the NHS over £40 million annually [3]. Moreover, there are significant health inequalities in the prevalence and severity of dental caries, with children from the most deprived areas experiencing more than twice the level of decay compared with those from the least deprived areas [2].

A key preventive measure is toothbrushing with a fluoride toothpaste [4]. Supplementing home‐based toothbrushing practices, supervised toothbrushing programmes (STPs) enable children, each day they attend nursery or school, to brush their teeth under staff supervision. Implemented worldwide, STPs have been found to be a cost‐effective [5] intervention in reducing dental caries and health inequalities [6, 7], and as such, in England, national guidance recommends the commissioning of STPs. However, uptake and maintenance are fragmented with considerable variation in how well they are implemented. Indeed, a recent survey [8], found that the current provision of STPs in England was low, with only half of the local authorities that responded implementing an STP. Nevertheless, with increased political interest in STPs [9, 10], and oral health now included in the statutory guidance for early years settings (e,g., nurseries) [11], primary and secondary schools [12], there is significant potential for an increase in the uptake of STPs.

Yet, to achieve maximum benefit, STPs must be adopted and sustained over time, but previous studies have highlighted several barriers and facilitators to the implementation of STPs [13, 14]. However, this research has largely focused on implementation at site level, and as such misses the wider determinants of implementation that occur at other levels of the implementation system, such as policymakers, oral health providers, parents and children. Moreover, this earlier research lacked an underpinning theoretical framework (with one notable exception [14]) and has argued that a stronger implementation science approach, utilising appropriate implementation frameworks is needed [13].

The Consolidated Framework for Implementation Research (CFIR) [15] has been recommended as the most robust means of exploring the barriers and facilitators to the implementation of STPs due to its comprehensiveness and utility, but as of yet this work has not been undertaken [13]. The CFIR, which has been applied to a variety of settings [15], is a practical meta‐framework of 39 constructs underlying five key implementation domains (see Table 1) that allows the systematic identification of the determinants of implementation to inform tailored strategies to improve implementation outcomes.

**TABLE 1:** Consolidated Framework for Implementation Research (CFIR) domain definitions.

CFIR domain	Definition
(1) Innovation	What is being implemented
(2) Outer setting	The wider context in which the innovation sits
(3) Inner setting	The place where the innovation will be implemented
(4) Individuals	The roles and characteristics of the individuals implementing the innovation
(5) Implementation process	The activities and strategies used to implement the innovation

Understanding the barriers and facilitators all stakeholders face in implementing STPs and the strategies they use to achieve success is critical to further understanding how to optimise, upscale and ensure the sustainability of STPs in the future. As such, this study aimed to explore the perspectives of key stakeholders on the barriers and facilitators at multiple levels of implementation of STPs using the Consolidated Framework for Implementation Research (CFIR).

## 2 Methods

This study employed qualitative research methods and is reported following the consolidated criteria for reporting qualitative research (COREQ) guidelines [16]. Ethical approval was provided by the University of Leeds Dental Research Ethics Committee (130 422/KGB/351).

### 2.1 Patient and Public Involvement and Engagement

Teachers, parents and children were included throughout the research process to ensure appropriateness of methods, topic guides and participant documentation. Regular meetings were held throughout the project with teachers representing mainstream and special education schools. A parent workshop was held within a local school to discuss the project, choose the design of the project logo, and provide advice on project documentation. Furthermore, creative methods were used to ensure the voice of the child was heard as the ultimate beneficiaries of supervised toothbrushing programmes, including a series of interactive storytelling and activity sessions with questions and discussion interwoven within the sessions in an age‐appropriate and engaging way.

### 2.2 Sample

Participants were stakeholders involved at different levels of STP implementation, including: (1) policymakers (representing health, education and sustainability) (2) providers of STPs (e.g. oral health promotion teams, dental practices, charities, social enterprise); (3) nursery/school staff; (4) parents/carers; and (5) children (aged 2‐6 years old). A purposive sample of stakeholders involved at various stages of STP implementation (ranging from never implementing an STP to running an established STP), with different socio‐demographic characteristics and models of funding and delivery for STPs were invited to participate from across England.

Professional stakeholders (i.e., policymakers, providers and nursery/school staff) were identified either directly or via advertisement through their networks. Parents/carers and children were recruited via senior management and staff within the nursery/school setting. All participants received an information sheet and consent form, with child‐friendly versions available. Written consent was obtained from all adult participants prior to the interview. For parent/carers written consent was obtained for their own participation and that of their child. For child participants written assent was obtained where possible with the support of parents/staff.

### 2.3 Data Collection

The CFIR version 1.0 [15] was used throughout data collection, analysis and interpretation, although elements of version 2.0 [17] were introduced during analysis as described below. Qualitative data was collected through semi‐structured individual interviews or focus groups (see Appendix S1 for details on research team and reflexivity) using an inductive‐deductive approach, with the topic guide designed to include open‐ended and CFIR‐focused questions.

Interviews with professional stakeholders and parents/carers were undertaken either face‐to‐face or on online platforms with only the interviewers and participants present. Before proceeding, interviewers introduced themselves and participants were allowed the opportunity to ask questions. Data collection took place between July 2022 and May 2024. Forty individual interviews and 17 (*n* = 119) focus groups were conducted with no repeat interviews. Interviews and focus groups lasted between 7 and 78 min (average 40 min). Field notes were made during and after interviews. All interviews were audio‐recorded, pseudo‐anonymised and transcribed verbatim. Transcripts were checked for accuracy by the interviewers but not returned to participants for checking. Data collection was iterative to data analysis, and recruitment continued until there was no new information that added to or shaped the overall interpretation of the data.

Parents/carers received a £15 voucher for their time. For nursery/school settings that recruited and consented to staff, parents/carers and children taking part in the study, a gift of £50 was provided to be used for the benefit of the children.

Research with young children requires flexibility and creativity in the methods used for data collection. To facilitate the engagement of children, participatory research methods were employed including interactive storytelling and the use of toys and puppets [16]. All sessions with children took place at their nursery/school and alongside the research team, were facilitated by nursery/school staff whose consent was obtained and their data included within the analysis.

### 2.4 Data Analysis

A deductive framework analysis [18] using an a priori thematic coding framework developed from the CFIR, the study aims, and familiarisation with the data was used to line‐by‐line code the transcripts. In parallel, an inductive approach was adopted to ensure openness to the identification of themes not defined by the coding framework. The NVivo software (Version 12, QSR International) was used for qualitative data handling. This provided retrieval facilities and coding remained connected to the original raw data throughout the refinement stages. Transcripts were coded independently (KG‐B, SE, KH, EL), with 10% of the transcripts being second coded [19]. Regular meetings were held to discuss coding, themes and resolve any discrepancies through discussion. Qualitative summaries of the coded data were developed across all framework domains, highlighting key thematic content and barriers and facilitators to the implementation of STPs. In addition, in line with CFIR coding guidelines each domain summary was given a rating of how positively or negatively it influenced implementation and the strength of this influence represented by + and − symbols (e.g. ++ Strong facilitator; − Weak barrier; + − Mixed). Due to the different form of data collection used with children, this data was analysed separately (SW) using thematic analysis (Appendix S2) and later combined with relevant CFIR themes after team discussion. Participants did not provide feedback on the findings of the study.

## 3 Results

The total sample after accounting for non‐participation (*n* = 2, stating time constraints) included 159 participants from across every region of England, consisting of: (1) policymakers (*n* = 24); (2) providers of STPs (*n* = 16); (3) nursery/school staff (*n* = 39); (4) parents/carers (*n* = 20); (5) children (*n* = 55; facilitators *n* = 5).

**FIGURE 1:**
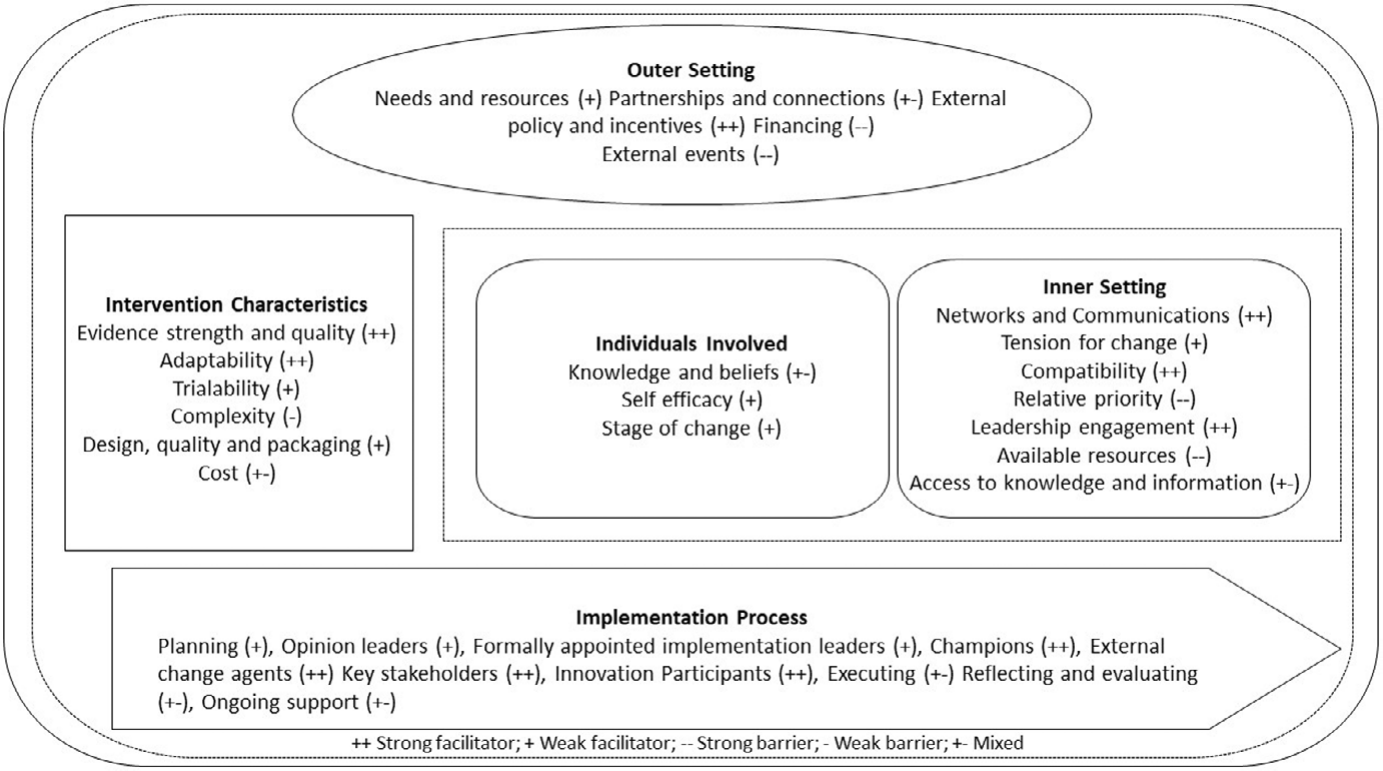
Strong and weak facilitators and barriers to implementation of supervised toothbrushing programmes by Consolidated Framework for Implementation Research (CFIR) domain.

Thirty‐five of the 39 CFIR 1.0 constructs were identified in the data. Three constructs derived from CFIR 2.0 were added to the coding framework (‘Financing’, ‘External events’, ‘Ongoing support’). ‘Partnerships and connections’ replaced ‘Cosmopolitanism’ as this more accurately reflected the data. The strongest facilitators to implementing STPs appeared in the ‘process of implementation’ domain, followed by the ‘intervention characteristics’ and ‘inner setting’ (see Figure 1 for an overview of the barriers and facilitators to STPs across the CFIR domains and underlying constructs).

Four themes were identified representing the key determinants of STP implementation: (1) Acceptability of STPs; (2) External ‘make or break’ conditions; (3) The importance of engagement across the system; (4) Desire for centralised support (Table 2). Each theme is discussed in turn with CFIR constructs italicised in parentheses alongside the strength/valence coding (see Table 3 for a summary of key thematic barriers and facilitators).

**TABLE 2:** Themes representing the key determinants of supervised toothbrushing programme implementation with associated Consolidated Framework for Implementation Research (CFIR) domains and constructs.

Theme	CFIR domains	CFIR constructs	Example quotes
1. Acceptability of supervised toothbrushing programmes	Intervention Characteristics, Inner Setting, Individuals Involved	Evidence strength and quality, Adaptability, Trialability, Design quality and packaging, Cost, Compatibility, Knowledge and Beliefs, Individual stage of change, Tension for change, Other personal Attributes, Complexity	‘*My practitioners are fully on board with it*.’ (Nursery Manager: Knowledge and beliefs). ‘*You do what works for you, you do what works in your area, it isn't a one‐size‐fits‐all*.’ (Oral health provider: Adaptability) ‘*I loved that idea. I think it's such an important skill. It's something that obviously we do in the morning and in the evening but sometimes feels like a little bit of just like a tickler for teeth with [a] toothbrush, and it is nice to think that there's that third toothbrushing situation going on in the day*.’ (Parent: Knowledge and beliefs) ‘…*giving the school the freedom to be able to fit it into their day themselves. Because I think from what I understood at one point was it was quite a manualised*, ‘*This is how you do it, and this is when you do it.’ And that didn't work for old schools*.’ (Policymaker: adaptability) *“And do you like brushing your teeth at nursery? I like it. I do it with [name of child].”* (Child responding to Facilitator: Compatibility)
2. External ‘make or break’ conditions	Outer Setting	External policy and incentives, Needs and resources, External events, Financing, Tension for change, Available resources	‘*I think it's really good that the government's put it into the Early Years Foundation Stage framework*.’ (Wider Context: External Policy and Incentives) ‘*I think it's worked well once it got off the ground, but then either funding runs out or you get a change of team, and then it all kind of folds*.’ (Policymaker: Financing). ‘…*at the height of the pandemic, you've got a lot of staff absences and things, nursery closures, and it was so difficult to keep a normal routine*.’ (Headteacher: External events).
3. The importance of engagement across the system	Outer Setting, Inner setting, Implementation Process	Partnerships and Connections, Key stakeholders, Leadership engagement, Relative priority, Opinion leaders, Formally appointed implementation leaders, Champions, External change agents	‘*But it is good though, because I think it sort of builds on that sort of partnership between us and the nursery as well in the sense that we are trying to do something at home and they're trying from their side as well*.’ (Parent: Key stakeholders) ‘*Because she's so good and passionate about it, and enthusiastic about these new things, it rolls down to the staff, which then rolls down to the children and then to the parents. And this is what we're saying is here is a prime example of it working perfect*.’ (Nursery Manager: Champion) ‘*I was the link person. I knew them all anyway because of my other role, which was useful knowing the people where I was working with and knowing the different types of settings that I was working with, so I think that's how that worked*.’ (Wider context: External change agent) ‘*Because I have a very small team, there's only three of us in my team, so we need that support and that partnership working from other agencies really to try and deliver oral health improvement programmes*.’ (Oral health provider: Partnerships and connections)
4. Desire for centralised support	Inner Setting, Individuals Involved, Implementation Process	Networks and Communication, Access to Knowledge and information, Planning, Self‐efficacy, Complexity	‘*But, you know, it would be really helpful to have, you know…this is a good way of doing it, this is what you need to look out for, basically do's and don'ts would be really helpful, things…don't let the children do this, and, you know, doing this would be really good idea*.’ (Childminder: Access to knowledge and information) ‘*But the problem is that people don't always know where to find things, and we've got so many different things, and then people want their badge on it, so then it has to be the local authority's version, so they start again because they think, oh, let's have a lovely new something. But actually, it's a terrible waste of resource because we're just all redoing and remaking. So, for me, I'd love it if we could develop and that's something we are looking at…is having a central point where all these things will be available*.’ (Policymaker: Access to knowledge and information) ‘…*any learning we can share about what's working well to keep this going I think would be really helpful*’. (Policymaker: Access to knowledge and information.) ‘*So, if there was a national agenda that needs to be an absolute protocol and that needs to be resourced for people to get properly trained*.’ (Policymaker: Access to knowledge and information) ‘*I think the best thing's if you had online resources, videos that showed them what to do*.’ (Oral health provider: Access to knowledge and information)

**TABLE 3:** Summary of the key barriers and facilitators to the implementation of Supervised Toothbrushing Programmes.

Barriers	Facilitators
Perceived burden	Strong evidence‐base
Funding constraints	Ability to tailor to local setting
Inadequate resources	External policy
COVID‐19	Engagement & partnership
	Strong leadership
	Planning
	Access to information & training

### 3.1 Theme 1: Acceptability of Supervised Toothbrushing Programmes

Stakeholders had a favourable perception of STPs, compelled by the strong scientific evidence of their effectiveness in reducing dental caries. This engendered high‐level support and funding from policymakers and uptake from nurseries/schools, particularly when parents and staff witnessed first‐hand the positive impact on children's health and development (*Evidence strength and quality ++*). Indeed, parents believed that undertaking toothbrushing with peers could develop children's toothbrushing skills and reduce their resistance to toothbrushing at home. Children enjoyed brushing their teeth at nursery/school alongside their friends. They described the process in detail (*Knowledge and beliefs + −*), including comparing it to toothbrushing at home.

Professional stakeholders felt strongly that the high prevalence of dental caries and the associated health and developmental impact on children was no longer tenable (*Tension for change +*). Professional stakeholders demonstrated high motivation and readiness towards promoting good oral health and a robust awareness of the oral health needs of children, especially those in deprived areas and with special educational needs and/or disabilities (*Needs and resources of those served +*). Consequently, they were open to STPs as it gave them a means of improving children's oral health (*Knowledge and beliefs + −*) and reducing inequalities (*Individual stage of change +*), with some nursery/school staff taking great pride in having a positive local impact with their STP. Although some felt STPs were burdensome or redundant, commitment to improving oral health tended to see them flourish over time.

Allowing nurseries/schools to tailor the STP to their specific needs helped sustain the programme long‐term and facilitated learning from different approaches (*Adaptability ++ and Trialability +*). It was important for STPs to fit with existing nursery/school routines (*Compatibility ++*), along with having a fun and child‐centred design for easy implementation (*Design, quality and packaging +*). However, there were mixed perceptions regarding costs, with concerns about initial setup costs, maintenance expenses and the time commitment from staff (*Cost + −*). Additional challenges included logistical issues, staff shortages and concerns about infection control (*Complexity −*). Despite these challenges, stakeholders generally believed that costs and complexities could be managed and offset once financing was secured.

### 3.2 Theme 2: External ‘make or break’ Conditions

This theme underscored the multifaceted nature of STP implementation within a complex publicly funded system, highlighting the interplay between policy, financing and resources. These ever‐evolving system factors, often beyond the control of stakeholders but with rippling effects, were able to ‘make or break’ STP implementation.

Oral health's inclusion in national statuary guidance for nurseries and schools was seen as a policy change that had boosted it as a priority (*External policy and incentives ++*). Despite piquing interest, as the guidance did not explicitly mandate STPs, and considering challenges regarding staff capacity, some nurseries/schools opted for one‐off oral health education approaches to meet this standard.

Funding constraints were one of the most significant barriers to STP implementation *(Financing −−)*, with the financial requirements for starting and sustaining an STP being a ‘huge’ factor that could stop the implementation of STPs irrevocably. At the policymaker level, obtaining funding to commission an STP was described as a slow and complicated process and due to changing funding mechanisms, an STP could ‘disappear overnight’. Decreased funding was felt to prohibit the time needed to work with nurseries/schools to tailor the STP to their needs, with these financial uncertainties cascading down to both providers and nurseries/schools. Despite these challenges, stakeholders strove to secure funding through various means, including partnerships with private entities, charities and parent contributions.

Inadequate resources relating to training, equipment and staff brought significant challenges for providers and nurseries/schools (*Available resources −−*). Low pay led to high staff turnover, which at the provider level created difficulties in providing comprehensive support and access to necessary training. At the nursery/school level, low staff numbers constrained staff time and ability to provide adequate support. The COVID‐19 pandemic exacerbated challenges, disrupting funding, resources and staff availability (*External events −−*).

### 3.3 Theme 3: The Importance of Engagement Across the System

Engaging key stakeholders within (*Partnerships and connections + −*) and across the system was an essential implementation facilitator (*Key stakeholders ++*). This ‘selling’ of an STP was integral to gaining collective ‘buy‐in’ and helped keep STP implementation a priority (*Relative priority −−*) when faced with other pressing public health concerns.

Ongoing, tailored engagement and support needed to be paired with sufficient local capacity, and as such professional stakeholders expressed strong views about the need for cross‐boundary collaborative working to drive STPs and promote learning (*Partnerships and connections + −*). Stakeholders reported how within and across implementation levels there were key individuals vital in influencing the uptake and maintenance of STPs by either providing leadership, serving as a ‘link person’ to other professionals and resources/information, championing STPs or advocating for change. These individuals drove implementation forward by ensuring collaboration and information‐sharing across boundaries. For instance, policymakers enabled change by campaigning for oral health programmes, including lobbying health boards, coordinating oral health across areas, and advocating for oral health within governing bodies. Some professionals influenced decisions regarding the uptake of STPs based on their experience and reputation (*Opinion leaders +*), whereas others were essential for the effective execution of STPs through engaging nursery/school staff to encourage motivation and ownership of the programme (*Formally appointed implementation leaders +*). Champions, most crucially within nurseries/schools (*Champions ++*), drove implementation by obtaining supplies, maintaining protocols and raising awareness amongst staff, parents and children.

Nevertheless, strong leadership engagement, particularly from senior leadership with the power to make final decisions and allocate staff were recognised as essential by all stakeholders in driving implementation forward (*Leadership engagement ++*). Policymakers emphasised the importance of receiving board‐level support in facilitating implementation, while within nurseries/schools leadership engagement was deemed vital to guide staff, parents, and children towards implementation. Effective leadership could facilitate or hinder implementation, with leaders dedicated to promoting STPs holistically, beyond just ‘brushing teeth’ being the most successful.

For nurseries/schools establishing and maintaining a strong partnership with parents was paramount. Extensive efforts were made to promote oral health to parents through multiple channels and approaches. However, parent engagement varied, influenced by background, beliefs and language barriers. Furthermore, while some concerns existed about potential reliance on the programme, both parents/carers and nursery/school staff largely welcomed STPs as a means of strengthening the learning between home and nursery/school. This was reinforced by the children who viewed the STP as part of their daily routine; talking about their home‐based toothbrushing in parallel, describing how they engaged with staff at nursery/school and their parents/carers during home toothbrushing.

### 3.4 Theme 4: Desire for Centralised Support

A key part of implementing STPs was having a sound plan (*Planning +*). Policymakers focused on strategic planning for oral health improvement, acquiring funding and awarding contracts. Providers emphasised ground‐level planning for organising supplies and engaging stakeholders, while nursery/school staff stressed the importance of clear action plans before daily implementation.

Ensuring stakeholders had access to guidance and support, particularly around the evidence‐base for STPs, examples of good practice, training, protocols and other documentation (e.g. consent forms) was seen as beneficial, but not always readily available (*Access to knowledge and information + −*). Although resources already existed, professional stakeholders were unaware of where to look for them, leaving them having to develop their own. Stakeholders were keen not to waste already stretched staff time ‘reinventing the wheel’, instead desiring a central repository to house relevant and consistent information and resources that could be adapted to their local context. A clear example of where such a central repository would be useful was training. All stakeholders expressed the importance of training in enhancing confidence in delivering STPs (*Self‐efficacy +*) but wished for this to be cascaded so that people across the organisation were ‘singing from the same hymn sheet’ (*Networks and communication ++*).

## 4 Discussion

This is the first study to explore the perspectives of key stakeholders involved in STPs at multiple levels of implementation (i.e., policymakers, providers, nursery/school staff, parents/carers and children) from across England. Numerous stakeholders with varying levels of interest, capacity and time, interacted (in varying and often unpredictable ways) to make STP implementation work, but they are situated within different levels of implementation and operated within their own systems.

Experiences were varied, but assessing the strength of stakeholder‐reported influences on the implementation of STPs elucidated several key determinants. STPs were perceived to be an acceptable and feasible option for promoting oral health. The intervention itself (i.e., toothbrushing with a fluoride toothpaste) is seemingly *simple* and most stakeholders saw them as relatively straightforward to implement and use, quite predictable in how they work and with few components. Other aspects were *complicated*; for example, seeking funding could involve multiple interacting parts, making it a difficult and lengthy process. Then there were aspects of implementing STPs perceived to be *complex*, such as the unpredictable, dynamic interactions between policy, financing and resources in the outer setting and their impact on implementation within the inner setting. As well as the various relationships across the system that were vital in driving implementation, but also highly unpredictable and difficult to disaggregate into constituent components with not all stakeholders having the capacity to plan, implement, monitor and adapt STPs or indeed engage with the system and build those all‐important partnerships and connections. Efforts to implement STPs can be greatly hindered by these complex systemic factors which are often beyond anyone's control. Previous work has shown that while public health programmes characterised by complicatedness are difficult but not impossible to implement, those characterised by complexity have a high chance of never becoming mainstreamed at all [20]. As such, this research highlights the conceptual complexity of STPs and speaks to the recent ‘complexity turn’ in public health research [21]; a recognition that one must consider system‐level effects and view interventions as complex system disrupters when implementing them [22, 23]. Therefore, further work is needed to either reduce the complexity of STPs or empower stakeholders to ‘run with’ the complexity [24]. Building relationships may be a good way for organisations/stakeholders in the system to do this, particularly when reducing complexity is not an option.

Organisational network analysis (ONA) is a method for studying communication and socio‐technical networks within organisations [25]. Based on social network theory [26], these types of analysis have identified that most organisations consist of two distinct and separate systems. The ‘formal’ system, (i.e., the visible and tangible elements of an organisation, such as its strategy, structure, processes, policies and values); and the ‘informal’ system, (i.e., the networks of relationships stakeholders form within and across groups, as they work to accomplish tasks together). This research builds on previous studies have highlighted the importance of relationships by reflecting similar ‘formal’ issues around funding, priorities, logistics and capacity; and ‘informal’ issues around communication, engagement, collaboration and empowerment across all levels of implementation [8, 13, 14]. Although traditional approaches to implementation have often discounted the role of ‘informal’ connections in bringing about change, implementation scientists are now increasingly recognising the importance of studying these ‘softer’ relational aspects of implementation more closely [27, 28, 29]. Indeed, the present findings suggest that while stakeholders must work within the requirements and provision of their ‘formal’ systems (e.g., funding, policy, available resources), it is their relationships in the overall system that enable adaptation in the face of funding cuts, problem‐solving in the face of unforeseen barriers, and promote trusting relationships and resilience to galvanise overall implementation efforts together and enable stakeholders to ‘run with’ the complexity they face.

Nevertheless, both systems could be better supported by having examples of good practice and credible resources readily available through a centralised hub. Across all levels, stakeholders wanted clear guidance on how to effectively implement an STP and despite there already being existing national guidance [2] and many locally developed resources, this information was not always easily accessible, of known quality, or up‐to‐date. Reflecting the wishes of stakeholders to not ‘reinvent the wheel’ and provide a centralised hub of quality‐assured, consistent information, the BRUSH (optimising toothbrushing pRogrammes in nUrseries and ScHools) project has co‐developed and piloted an online implementation toolkit (www.supervisedtoothbrushing.com) that brings together new and existing resources to help support the implementation of supervised toothbrushing programmes at all levels of implementation.

For STPs ongoing engagement certainly seemed to help weather the storm of complex implementation challenges. However, other strategies that may help stakeholders deal with complex STP implementation include strengthening leadership, providing more central support, co‐developing a collective vision of STPs with all stakeholders, developing individuals, using their creativity as a resource and improving the policy context. Future work may benefit from applying complexity science principles and systems thinking to future evaluations and research around STP implementation. This is because the value of quantifying determinants of implementation is questionable as different influences will play out very differently depending on circumstances. Papoutsi, Greenhalgh & Marjanovic (2024) [30] provide a useful overview of frameworks, models and theories which explain spread, scale up and sustainability. Within this they identified three frameworks which see spread, scale up and sustainability as dynamic processes occurring within complex systems marked by uncertainty, unpredictability and emergent properties. One of these frameworks is the NASSS framework (Non‐adoption, Abandonment, Scale‐up, Spread and Sustainability) [31]. Although developed to understand the implementation of technological interventions it would be interesting to use a framework like this to understand which aspects of STP implementation are complex, how might this complexity be reduced, and how might individuals, sites and organisations be supported to handle the remaining complexities better.

A limitation of the current study was that the interviewed stakeholders were already aware of STPs and held strong opinions about them, whether that be positive or negative, therefore the perspectives of those more indifferent towards STPs may not have been captured. Even so, a range of barriers and facilitators to implementation were identified covering all five CFIR domains. A key strength was the inclusion of stakeholders at different levels of implementation and different geographic areas of England, including children whose insights are often seldom heard [32]. Another strength was the use of the CFIR, which ensured the research was theoretically informed with a clear focus on how to facilitate more effective future implementation.

In conclusion, this is the first study to qualitatively explore the barriers and facilitators to STP at multiple levels of implementation underpinned by an implementation science framework. The findings have strong implications for policymakers who wish to implement STPs, for although a seemingly simple intervention, with strong support from a variety of invested parties, the implementation of STP is complex with a range of barriers and facilitators requiring consideration at multiple levels of implementation. Support to establish and maintain an STP was requested by all stakeholders. In response, this work has facilitated the co‐design and piloting of a supervised toothbrushing implementation toolkit, which provides a central hub of resources and good practice to optimise implementation of STPs at scale.

## Author Contributions

K.G.‐B., Z.M., P.D., K.H. and S.E. contributed to the conception and design of the study. K.G.‐B., S.E., E.L., Z.M., S.W. and H.E. contributed to data collection. K.G.‐B., S.E., E.L., K.H., S.W. contributed to the analysis and interpretation of the study. All authors drafted, critically revised and approved the final manuscript.

## Ethics Statement

Ethical approval was provided by the University of Leeds Dental Research Ethics Committee (130 422/KGB/351).

## Conflicts of Interest

The authors declare no conflicts of interest.

## Supporting information


Appendix S1.

Appendix S2.

## Data Availability

A matrix of all the CFIR findings across all stakeholders with scores and summaries is available upon request.
