# Supplementary material for: Supervised Toothbrushing Programmes: Understanding Barriers and Facilitators to Implementation
Source: Community Dent Oral Epidemiol. 2025 Jan 29;53(3):256–64. doi: 10.1111/cdoe.13026 (PMC12064875; doi:10.1111/cdoe.13026)
Supplement: Supplementary file 1 — Appendix S1. [file CDOE-53-256-s001.docx]

*Research team and reflexivity*

KG-B (female) is a chartered psychologist and lecturer with a PhD. SE (female) is a qualified dentist and research associate with a PhD. KH (male) is an implementation specialist, with a PhD covering implementation science and has significant experience of designing and carrying out implementation research within public health settings. ZM (female) is a professor and honorary consultant in dental public health and a qualified dentist with a PhD. SW (female) is a qualified dentist currently completing specialist training in dental public health. EL (female) is a research administrator with a background in art history. KG-B, SE, KH, ZM and SW have experience in qualitative research The interviewers all have an interest in improving children’s oral health and had no relationships with the participants prior to initial contact except for some of the professional stakeholders who were in the same professional circle.
