# Supplementary figures and images for: Supervised Toothbrushing Programmes: Understanding Barriers and Facilitators to Implementation
Source: Community Dent Oral Epidemiol. 2025 Jan 29;53(3):256–64. doi: 10.1111/cdoe.13026 (PMC12064875; doi:10.1111/cdoe.13026)

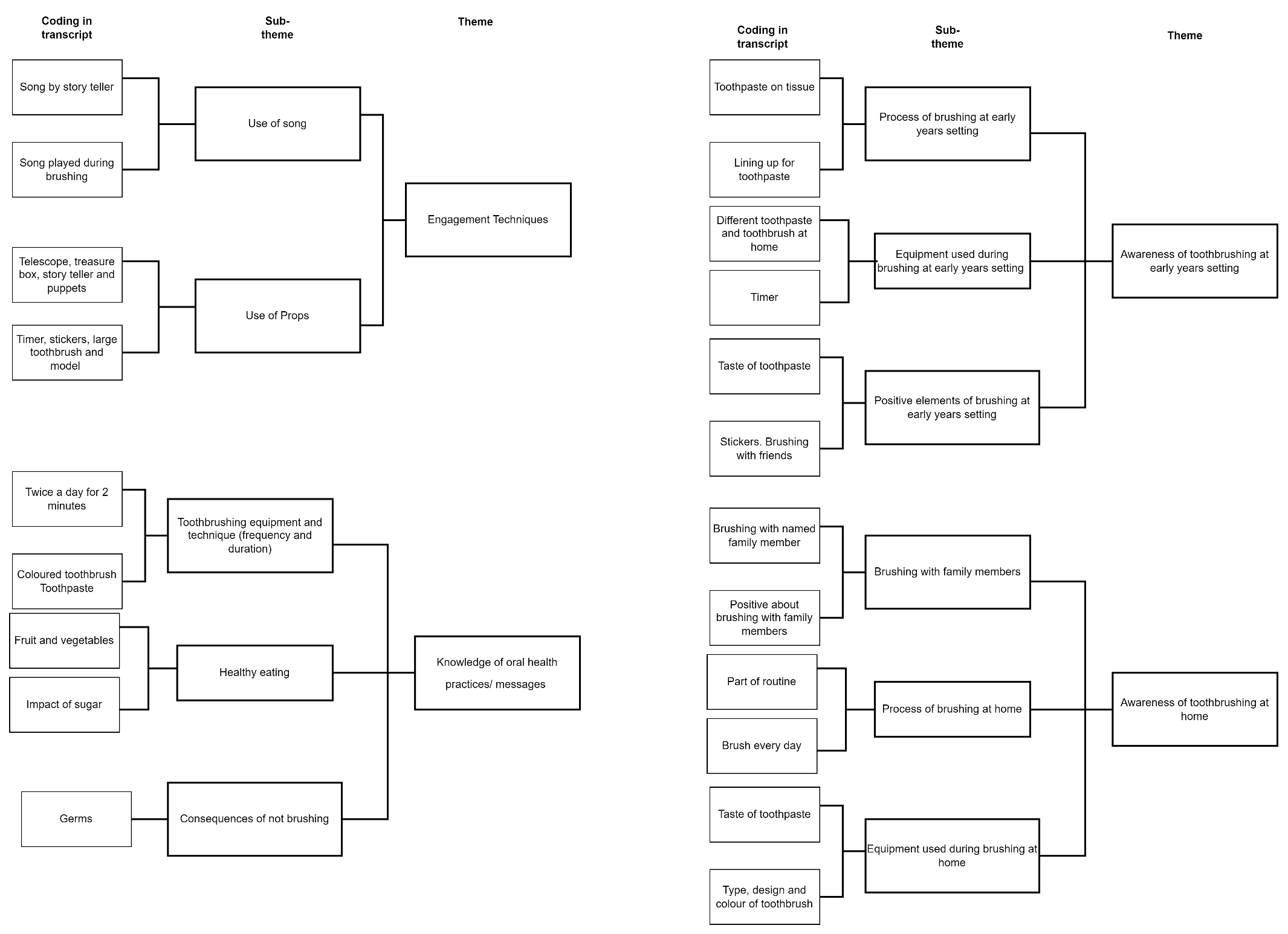

Supplement: Supplementary file 2 — Appendix S2. [file CDOE-53-256-s002.docx]
